# Diffuse alveolar hemorrhage in a pediatric patient with cri du chat syndrome: a case report

**DOI:** 10.1097/MS9.0000000000005072

**Published:** 2026-06-15

**Authors:** Rizuna Sharma, Dhiraj Adhikari, Bibek Shrestha, Sudeep Kc, Pradeep Gupta, Suraj Aryal

**Affiliations:** a Department of Pediatrics, Tribhuvan University Teaching Hospital, Kathmandu Nepal; b Maharajgunj Medical Campus, Tribhuvan University, Institute of Medicine, Kathmandu Nepal

**Keywords:** cri du chat syndrome, diffuse alveolar hemorrhage, genetic disorder, pediatric pulmonary hemorrhage

## Abstract

This case highlights a novel association between cri du chat syndrome (CdCS) and diffuse alveolar hemorrhage (DAH), a life-threatening pulmonary complication not previously reported in the literature. It emphasizes the importance of considering DAH in pediatric patients with genetic disorders presenting with recurrent hemoptysis and anemia. Early recognition, comprehensive evaluation, and multidisciplinary management are crucial for improving outcomes. This report expands the clinical spectrum of CdCS and urges heightened awareness among pediatricians and pulmonologists.

## Introduction

Cri du chat syndrome (CdCS), also known as 5p-syndrome, is a rare genetic disorder caused by a deletion on the short arm of chromosome 5. It is clinically characterized by a high-pitched, catlike cry, microcephaly, distinctive facial features, and severe developmental delay^[^1^]^. Children with CdCS often experience frequent hospitalizations, with respiratory illnesses being a major contributor to morbidity and mortality^[^2,3^]^.

Diffuse alveolar hemorrhage (DAH) is a rare but potentially life-threatening pulmonary condition that typically presents with hemoptysis, anemia, and radiographic evidence of diffuse alveolar infiltrates^[^4–6^]^. DAH can be caused by various conditions, including vasculitis, infections, and coagulopathies, but its occurrence in patients with genetic syndromes is uncommon^[^7,8^]^. To our knowledge, there have been no prior reports documenting an association between CdCS and DAH. In this case report, we present an 18-month-old female child with genetically confirmed CdCS who developed recurrent hemoptysis, anemia, and radiological features consistent with DAH. This report highlights a novel clinical manifestation of CdCS and underscores the need for increased awareness of pulmonary hemorrhage as a potential complication in such patients.

HIGHLIGHTS

- This case highlights a novel association between cri du chat syndrome and diffuse alveolar hemorrhage (DAH), a life-threatening pulmonary complication not previously reported in the literature.
- It emphasizes the importance of considering DAH in pediatric patients with genetic disorders presenting with recurrent hemoptysis and anemia.
- Early recognition, comprehensive evaluation, and multidisciplinary management are crucial for improving outcomes.
- This report expands the clinical spectrum of cri du chat syndrome and urges heightened awareness among pediatricians and pulmonologists.

## Case history/Examination

**Figure 1.**
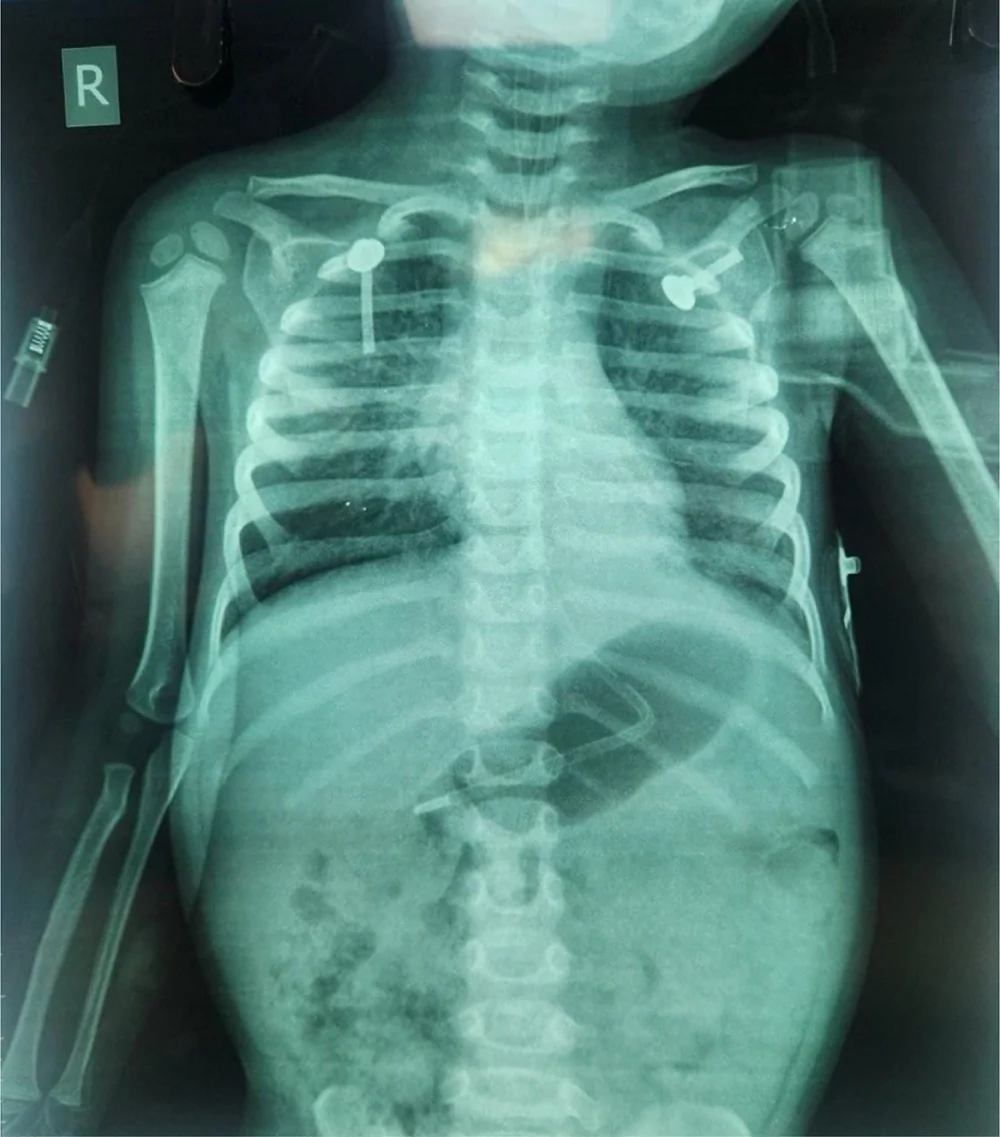
Chest X-ray posteroanterior view showing haziness in bilateral lung field.

**Figure 2.**
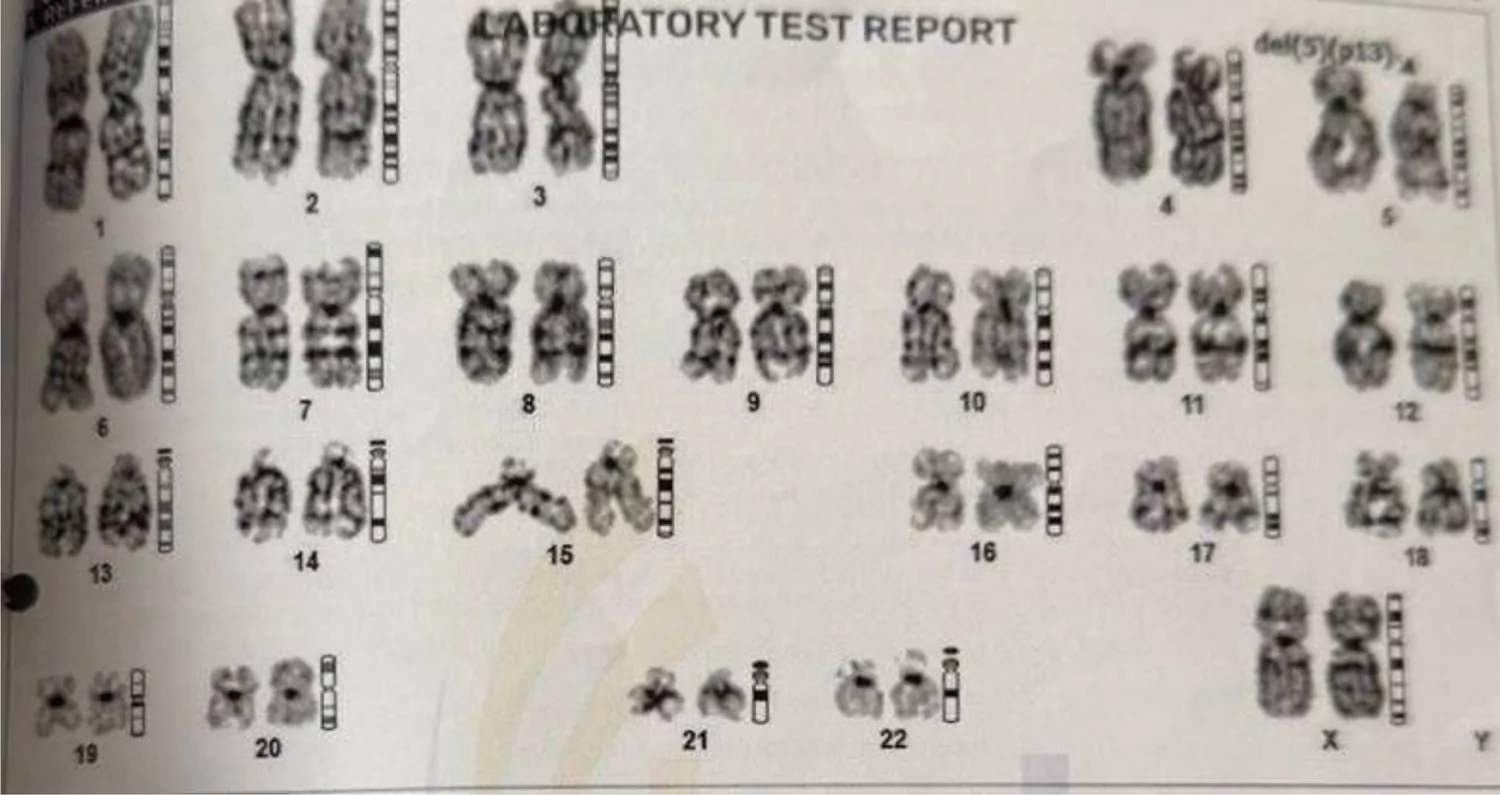
Karyotyping of the child.

**Figure 3.**
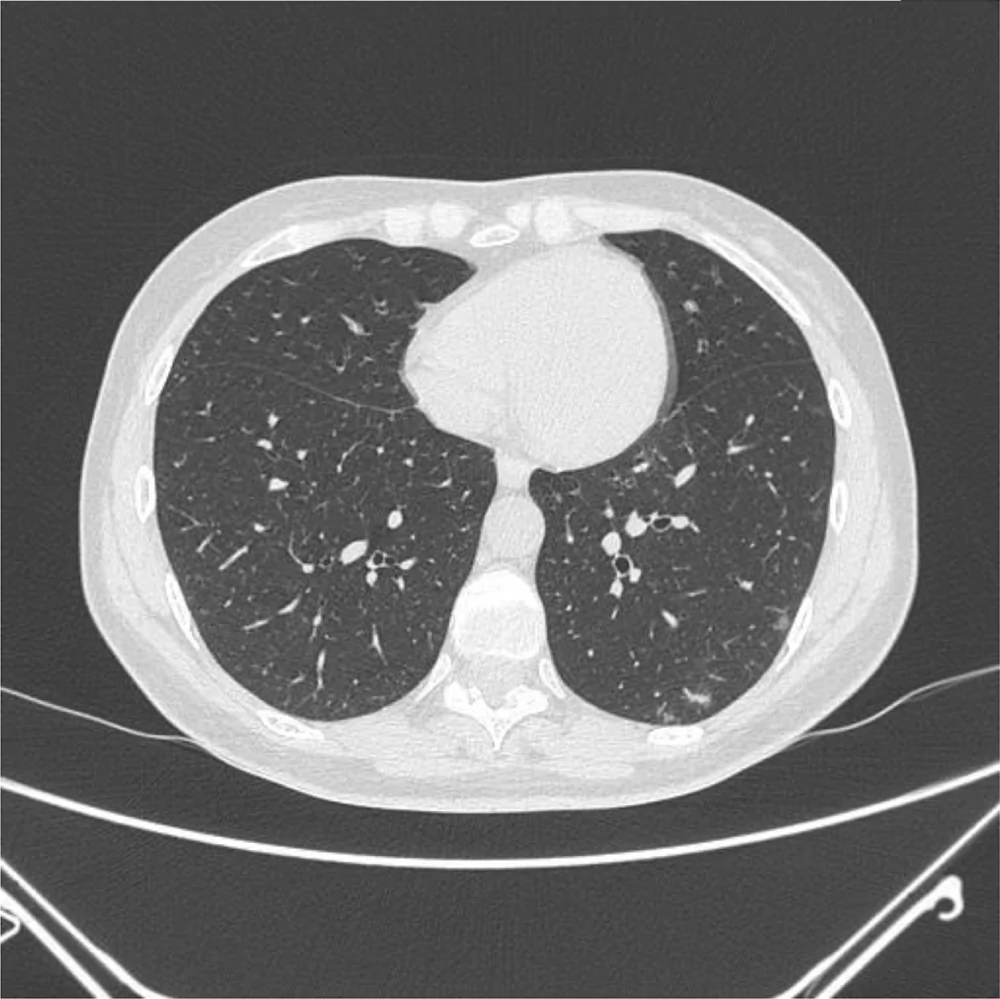
HRCT chest showing consolidation with air bronchogram and adjacent ground glass opacities in bilateral lung fields.

An 18-month-old female child presented to our hospital with coughing up blood-mixed sputum and fast breathing for 5 days. She was the firstborn to non-consanguineous parents, with no significant perinatal history. At 3 months of age, she presented with fever and cough and was treated for pneumonia with intravenous (IV) antibiotics for 1 week at her hometown hospital. She remained asymptomatic for 2 months thereafter. Subsequently, she presented with fever, cough, and noisy breathing, similar to her previous episodes, and was treated with IV antibiotics. Echocardiography was done to rule out any congenital heart disease, and nasopharyngeal laryngoscopy (NPL) was performed, which detected laryngomalacia. At 6 months of age, she presented with similar symptoms to our hospital, along with a typical catlike cry and hypotonia. All investigation parameters were normal except for a hemoglobin level of 10 g/dl. Further hematologic workup was planned but could not be performed because the patient’s family could not afford it. An X-ray showed haziness in the bilateral lung fields, so the baby was treated for pneumonia with IV antibiotics for 7 days (Fig. 1). To rule out any airway malformation, NPL and magnetic resonance imaging of the neck were performed, both of which were normal. Genetic testing was advised to rule out any syndromic association, but the parents refused. Then, 4 months later, at 10 months of age, she was taken to a different hospital for a similar illness, treated for pneumonia, and sent home. At 12 months of age, her presentation was atypical compared to before, as she presented with blood in sputum and increasing paleness for the first time, along with delays in all growth and developmental milestones. Her investigations, sent on an outpatient basis, showed hemoglobin (Hb) of 11.7 g/dl. Peripheral blood smear revealed microcytic hypochromic red cells with a few pencil cells. Serum iron was 16 µg/dl, total iron-binding capacity (TIBC) was 483 µmol/L, transferrin saturation was 3%, and ferritin was 73 µg/l. She was treated for iron deficiency anemia. However, a month later, she presented with a similar complaint to a local hospital, where her hemoglobin level had dropped to 7.2 g/dl. Hemoglobin electrophoresis was performed, which was normal, and she was treated with IV antibiotics. She also received a blood transfusion for the first time during this period. Thereafter, until 15 months of age, she was admitted 2–3 times for the same issue and received blood transfusions during each visit. At 15 months of age, she was taken to a city hospital for fever, coughing up blood in sputum, and increasing paleness. Her hemoglobin was found to be 3.3 g/dl, so she received blood products. Karyotyping was performed due to her dysmorphic features, and bone marrow analysis revealed a deletion in the 5p chromosome, suggestive of CdCS (Fig. 2). The child was kept on antibiotics for 5 days and sent home. Three 3 months later, at 18 months of age, she presented with similar symptoms as before. On evaluation, her hemoglobin had dropped to 5.5 mg/dl, so blood products were transfused. For recurrent blood in sputum, high-resolution computed tomography (HRCT) of the chest was performed, which showed consolidation with air bronchogram and adjacent ground-glass opacities in the bilateral lung fields, suggestive of acute respiratory distress syndrome (ARDS)/pulmonary hemorrhage (Fig. 3). Furthermore, a CT aortogram was performed to check for structural anomalies, which was found to be normal. Tuberculosis workup was also normal, and the child was then referred to our center. At our hospital, the child was dysmorphic, hypotonic, and pale. There was no lymphadenopathy, cyanosis, or rash. As the child was not in severe respiratory distress and oxygen saturation was maintained on room air, she was admitted to the ward for further evaluation. On anthropometric evaluation, her weight, height, and head circumference were below average. On systemic evaluation, there was bilateral symmetrical chest rise with equal air entry and bilateral diffuse crepitation. Other systems showed no pathology.

## Differential diagnosis

**Figure 4.**
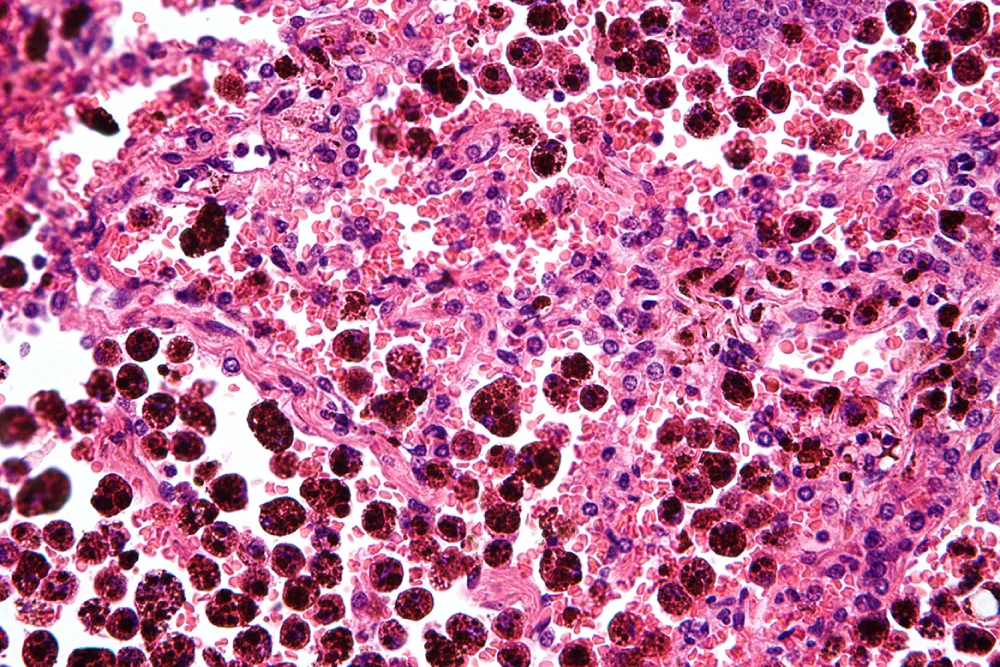
Lung biopsy showing intra-alveolar hemorrhage and hemosiderin-laden macrophages.

During her stay in the ward, septic markers were negative as before. X-ray showed diffuse heterogeneous opacities (Fig. 1). Vasculitis workup, along with genetic testing, was planned. However, on the third day of admission, she developed ARDS and was in severe respiratory distress. Consequently, she was admitted to the Pediatric Intensive Care Unit (PICU), intubated, ventilated, and given a blood transfusion. She was planned for further workup of pulmonary hemorrhage. During her stay in the PICU, broncho alveolar lavage was performed, which was negative for heart–lung malfunction and malignancy. Vasculitis workup [ANA (ELISA): 51.7 AU/mL, P-ANCA: 22 AU/mL (low positive), C-ANCA: 12 AU/mL] was also low positive, so it was considered false positive. Lung biopsy was performed, which showed variable-sized air spaces with intra-alveolar hemorrhage and hemosiderin-laden macrophages (Fig. 4). Echocardiography was conducted to rule out any congenital heart disease, and it was normal. Other diagnostic workups, including the immunoglobulin profile, anti-phospholipid antibody, and anti-GBM antibody, could not be performed due to financial issues. In this case, DAH warranted exclusion of key etiologies. Autoimmune causes, including ANCA-associated vasculitis, were ruled out via negative serologic testing and the absence of renal involvement. Infectious etiologies, such as bacterial or viral pneumonia, were deemed superimposed aggravating factors secondary to a primary unidentified cause. Drug-induced DAH was excluded through a detailed medication history revealing no anticoagulant or chemotherapeutic exposure. Coagulopathies were dismissed with normal platelet counts and coagulation profiles. Imaging findings of diffuse heterogeneous opacities without focal lesions and bronchoscopy confirming progressively bloody lavage supported DAH, while histopathology excluded rare mimics like capillary hemangiomatosis. This systematic exclusion narrowed the diagnosis to DAH, likely secondary to CdCS.

## Conclusion and results (outcome and follow-up)

The patient was treated with an injection of methylprednisolone at 30 mg/kg/day for 5 days and then transitioned to oral prednisolone at 2 mg/kg/day for 4 weeks, followed by a tapering dose. On the 10th day of the PICU stay, the child developed new-onset pneumonia/ventilation-associated pneumonia (VAP), after which empirical antibiotics colistin and levofloxacin were added. Subsequently, a mini-bronchoalveolar lavage (BAL) was performed, which isolated two organisms: *Pseudomonas* and *Klebsiella*. As a result, targeted therapy with ceftazidime and amikacin was initiated. The first dose of cyclophosphamide was administered, after which the child showed clinical improvement. ARDS gradually became less severe, and an extubation trial was attempted after 14 days of ventilation. However, it was not tolerated due to severe subglottic stenosis and unresolved VAP, necessitating re-intubation. In the third week, the child was on full feeds, and cotrimoxazole and fluconazole were added due to the prolonged PICU stay. During the fourth week, another extubation trial was attempted, but it was unsuccessful due to pooling of secretions and upper airway obstruction. Consequently, a tracheostomy was performed, and gradual weaning from ventilation via the tracheostomy was attempted over the following week. In the sixth week of the hospital admission, the child developed new-onset pulmonary hemorrhage with shock. She was placed on high ventilation parameters and vasoactive support but succumbed to a morbid state due to a hospital-acquired infection. The patient’s family was informed about the prognosis, and they opted for the withdrawal of life support.

This case challenges the typical view of CdCS, primarily involving neurological, cardiac, and craniofacial abnormalities. It underscores the importance of considering DAH in CdCS patients who present with acute respiratory decline with hemoptysis. Early bronchoscopy and BAL are crucial for diagnosis, as hemorrhage may not always be immediately obvious without these diagnostic tools. DAH can present similarly to aspiration pneumonia or respiratory infections in CdCS patients, and when hypoxia is more severe than expected based on radiographic findings, it should prompt consideration of hemorrhage. Moreover, a collaborative, multidisciplinary approach involving genetics, pulmonology, and speech therapy is vital for addressing complex issues such as aspiration risk, immune function, and rehabilitation needs, ensuring comprehensive care for such patients. This case report has several limitations that must be considered while interpreting the findings. First, while clinical history strongly suggested chronic aspiration as a contributor, aspiration was not confirmed through video fluoroscopy, which could have provided more definitive evidence. Additionally, whole exome sequencing was not performed, meaning potential genetic modifiers, such as unrecognized variants in coagulation or immune-related genes, could have influenced the patient’s phenotype. The single-case nature of the report limits the ability to establish causality, and larger cohorts would be needed to confirm the findings. This case emphasizes the need for further research into potential complications of CdCS, particularly DAH, which may arise from vascular fragility, immune dysregulation, or chronic aspiration. Future genetic studies targeting 5p genes, such as *CTNND2*, in CdCS patients with pulmonary hemorrhage could provide valuable insights into underlying mechanisms. Immune profiling through flow cytometry or cytokine panels may help identify dysregulated immune pathways contributing to respiratory complications. Additionally, population studies and surveillance in CdCS registries will be crucial for determining the incidence of DAH. This case underscores the importance of maintaining a high clinical suspicion for DAH in CdCS patients presenting with respiratory symptoms, as early detection and intervention could be lifesaving. Documenting such rare associations is essential for refining care strategies and improving outcomes for patients with genetic syndromes.

## Discussion

Respiratory complications are frequent in CdCS, with neonatal respiratory distress occurring in some infants, and about 30% of children with the condition experiencing pneumonia, bronchitis, and otitis media^[^2^]^. In our case, an 18-month-old child with CdCS presented with symptoms of recurrent fever, cough, and hemoptysis; was progressively anemic and was concluded to have DAH. The novel association of DAH with CdCS in this pediatric patient underscores the importance of synthesizing clinical history, targeted investigations, and multidisciplinary management in rare genetic disorders.

CdCS is caused by a deletion in the 5p15.2 region of chromosome 5, which contains genes vital for both neurodevelopment and vascular function. One such gene, *CTNND2* (catenin delta-2), located in this region, is crucial for cell-cell adhesion and the stability of blood vessels^[^3^]^. A reduction in *CTNND2* function could compromise vascular integrity, making alveolar capillaries more susceptible to rupture under conditions of stress, such as infection or inflammation. Although vascular abnormalities are not typically linked with CdCS, this case suggests the potential for a previously unidentified pulmonary vascular involvement. Children with CdCS frequently show immune dysfunction, such as IgA deficiency and recurring respiratory infections^[^8^]^. While our patient’s autoimmune tests were negative, any subtle immune imbalances could still contribute to localized alveolar inflammation. For instance, impaired T-cell activity or cytokine production (e.g., IL-6, TNF-α) may intensify damage to alveolar capillaries, even without evidence of widespread autoimmunity. Moreover, hypotonia and swallowing difficulties, which are common in this syndrome, heighten the risk of chronic silent aspiration^[^9^]^. Repeated micro aspirations may lead to subclinical pneumonitis, damaging alveolar membranes and ultimately resulting in hemorrhage. The presence of hemosiderin-laden macrophages in our patient provides histopathological evidence of previous bleeding episodes, which could be associated with chronic aspiration. A comprehensive diagnostic workup effectively ruled out common causes of DAH in this patient. Autoimmune conditions were excluded due to negative serologies for ANCA and ANA, along with the absence of renal involvement, which dismissed the likelihood of vasculitis or Goodpasture syndrome. Cardiac causes were considered and ruled out through echocardiography, which confirmed no pulmonary edema linked to congenital heart defects, such as ventricular septal defects–a condition seen in 15-20% of CdCS patients^[^10^]^. These findings collectively reinforce the hypothesis that the DAH in this patient is likely connected to the inherent vulnerabilities associated with CdCS. In children with CdCS, respiratory issues are often linked to chronic aspiration and hypotonia, which can delay the consideration of other conditions. Primary ciliary dyskinesia (PCD), a rare genetic disorder with similar respiratory symptoms, may also be present in CdCS patients^[^11^]^. PCD is caused by mutations in several genes, including DNAH5, which accounts for about 30% of cases. Interestingly, DNAH5 is in the same 5p region affected in CdCS, raising the possibility that some CdCS patients may also have PCD. This overlap underscores the importance of considering PCD in the differential diagnosis of respiratory problems, such as DAH, in CdCS patients. The management of DAH in this patient required a tailored approach that addressed both acute stabilization and the unique complexities of CdCS. Supportive care included high-flow nasal cannula to improve oxygenation^[^1^]^ and packed red blood cell transfusions to correct anemia, with careful fluid management due to the patient’s borderline pulmonary hypertension. Although methylprednisolone pulse therapy was initiated empirically to address potential immune-mediated alveolar inflammation^[^12^]^, it was unresponsive in this case, indicating that the inflammation may not have been steroid-sensitive. To mitigate aspiration, postural adjustments were used to reduce recurrent alveolar injury^[^13^]^. Additionally, chest physiotherapy aided in secretion clearance, and family counseling emphasized the importance of monitoring respiratory symptoms^[^14^]^, given the novel association between CdCS and DAH. Despite aggressive supportive and immunosuppressive therapy, the patient’s clinical course was marked by rapid deterioration, resulting in mortality.

## Data Availability

None.

## Patient perspective

At first, we thought our child was just having another chest infection – she’d always been prone to coughs and colds. But this time, something felt different. Her breathing became rapid and shallow, like she was fighting for every breath. We kept asking, “Why isn’t this getting better?” but no one had answers. It was terrifying to watch her struggle and not know how to help. When the doctors mentioned a rare lung complication, we were shocked. We’d never heard of DAH before, let alone a link to cri du chat syndrome. The team explained it was new to them too, but they worked tirelessly to find the cause. The bronchoscopy and ventilation support scared us a lot. Eventually, after a tiresome effort and a not-so-hopeful prognosis, we opted to withdraw her life support. Now, we want other families like ours to know trust your instincts. If something feels wrong, push for answers. We hope our child’s story helps doctors recognize DAH earlier in kids like ours. Please, share this knowledge – it might save a life.

## References

[1] Chehimi SN Zanardo ÉA Ceroni JRM. Breakpoint delineation in 5p- patients leads to new insights about microcephaly and the typical high-pitched cry. Mol Genet Genomic Med 2019;8:e957. 31568707 10.1002/mgg3.957 PMC7005617

[2] Schrier SA Sherer I Deardorff MA. Causes of death and autopsy findings in a large study cohort of individuals with Cornelia de Lange syndrome and review of the literature. American J Med Genet Part A 2011;155:3007–24. 10.1002/ajmg.a.34329 PMC3222915 22069164

[3] Spoto G Accetta AS Grella M. Respiratory comorbidities and complications of cerebral palsy. Dev Neurorehabil 2024;27:194–203. 38992903 10.1080/17518423.2024.2374959

[4] Panikkath D Gadwala S Mills B. Diffuse alveolar hemorrhage. Southwest Respir Crit Care Chron 2015;3:19.

[5] Baumgartner M Ashton R. Diffuse alveolar hemorrhage. South Med J 2011;104:274–75. 21606696 10.1097/SMJ.0b013e3182126d6d

[6] Newsome BR Morales JE. Diffuse alveolar hemorrhage. South Med J 2011;104:269–74. 21606695 10.1097/SMJ.0b013e3182126d3b

[7] Picard C Parrot A Mayaud C. Hémorragiesalvéolaires en dehors des situations d’immunodépression: prise en charge diagnostique et thérapeutique. La PresseMédicale 2009;38:1343–52. 10.1016/j.lpm.2008.07.008 19446997

[8] Corrêa T Feltes BC Riegel M. Integrated analysis of the critical region 5p15.3–p15.2 associated with cri-du-chat syndrome. Genet Mol Biol 2019;42:186–96. 30985858 10.1590/1678-4685-GMB-2018-0173 PMC6687350

[9] Dayman R Wilson AC Blackmore AM. Healthcare usage for respiratory illness by paediatric inpatients with cerebral palsy. ActaPaediatrica 2020;110:1554–55. 10.1111/apa.15658 33171532

[10] Hills C Moller JH Finkelstein M. Cri du chat syndrome and congenital heart disease: a review of previously reported cases and presentation of an additional 21 cases from the pediatric cardiac care consortium. Pediatrics 2006;117:e924–7. 16585274 10.1542/peds.2005-1012

[11] Shapiro AJ Weck KE Chao KC. Cri du chat syndrome and primary ciliary dyskinesia: a common genetic cause on chromosome 5p. J Pediatr 2014;165:858–61. 25066065 10.1016/j.jpeds.2014.06.048 PMC4177261

[12] Susarla SC Fan LL. Diffuse alveolar hemorrhage syndromes in children. Curr Opin Pediatr 2007;19:314–20. 17505192 10.1097/MOP.0b013e3280dd8c4a

[13] Vece TJ De Guzman MM Langston C. Diffuse alveolar hemorrhage in children. Kendig’s Dis Respir Tract Child Elsevier 2019:893–902.e2. doi:10.1016/B978-0-323-44887-1.00061-4.

[14] Cohen SP Eisner M Fussner LA. Diffuse alveolar hemorrhage in pediatrics: etiologies and outcomes. Pediatr Pulmonol 2024;59:3364–70. 39115444 10.1002/ppul.27207 PMC11601033

